# Chromosomal analysis of single sperm cells from infertile couples with severe oligoteratozoospermia: A cross-sectional prospective study

**DOI:** 10.1371/journal.pone.0303350

**Published:** 2024-06-14

**Authors:** Supitcha Sassanarakkit, Sudaporn Chamnankran, Artitaya Singwongsa, Matchuporn Sukprasert, Chonthicha Satirapod

**Affiliations:** Department of Obstetrics and Gynecology, Faculty of Medicine, Ramathibodi Hospital, Mahidol University, Bangkok, Thailand; University of Rijeka Faculty of Medicine: Sveuciliste u Rijeci Medicinski fakultet, CROATIA

## Abstract

In this cross-sectional prospective study, advanced next-generation sequencing technology was used to compare the molecular karyotyping of individual human sperm cells in infertile couples with severe oligoteratozoospermia (i.e., low sperm count and motility) to those of infertile couples with normal semen. Fourteen infertile couples who were patients at Ramathibodi Hospital in Bangkok, Thailand, were recruited from January to November 2023, and they were categorized into two groups based on semen analysis results. The study group comprised couples with severe oligoteratozoospermia, whereas the control group exhibited normal semen. Individual sperm cells from the semen samples were isolated by the micromanipulation technique for subsequent whole-genome amplification and next-generation sequencing, where the primary outcome was the aneuploidy rate. Seventy individual sperm cells were isolated with a 90% success rate for amplification. The next-generation sequencing results showed that the aneuploidy rate was 25%–75%, with a mean of 48.28% in the study group. In contrast, the control group exhibited aneuploidy rates of 0–75%, with a mean of 15.15%. The difference between the two groups was statistically significant (odds ratio: 5.8, 95% confidence interval: 1.30–26.03). Sperm cells of the study group showed a threefold higher aneuploidy rate than those in the control group, even though the sperm cells were selected by micromanipulation for their normal morphology. Comprehensive counseling is recommended to address elevated aneuploidy rates that potentially surpass those of the general infertile population. Guidance on preimplantation genetic testing is also recommended to ensure the transfer of embryos with normal chromosomes.

## Introduction

In 1978, the birth of the first test-tube baby marked a breakthrough in infertility treatment and assisted reproductive technology (ART), which is now used around the world to treat infertility resulting from a broad spectrum of medical conditions. ART comprises various stages, including ovarian stimulation with hormones, oocyte pick-up, fertilization, and embryo transfer. ART has helped with the birth of more than 10 million infants worldwide, and the clinical use of the stimulation cycle is increasing annually, resulting in approximately 500,000 children being born via IVF each year [1, 2]. The main fertilization techniques are in vitro fertilization (IVF) and intracytoplasmic sperm injection (ICSI), of which the latter was developed later to combat severe male factor infertility. The utilization of the ICSI process has continuously increased as the number of indications for its application has grown [3]. The 2014 report by the International Committee for Monitoring Assisted Reproductive Technologies (ICMART) indicates a global inclination toward choosing ICSI over IVF [4]. Recent studies conducted in Europe and Japan revealed that ICSI is still the preferred method in ART treatment cycles and is used twice as often as traditional IVF in registered cycles [3, 5].

ICSI primarily involves selecting sperm with normal morphology and motility to fertilize a metaphase II oocyte. ICSI relies on the expertise of an embryologist, who uses the micromanipulation technique to select only the most suitable sperm. A micromanipulation is a cost-effective approach for isolating individual sperm cells. Still, uncertainty remains over whether the selection process can definitively eliminate all genetic defects in sperm within a group with highly abnormal semen parameters, especially in cases of severe oligoteratozoospermia (i.e., low sperm count and motility) [6]. Although some studies have proposed intracytoplasmic morphologically selected sperm injection (IMSI) to select the best sperm to achieve optimal pregnancy outcomes, this technique is still under investigation [7]. Many genetic factors have been implicated in male infertility, and prior studies have consistently demonstrated that individuals experiencing severe oligozoospermia (i.e., low sperm count) and teratozoospermia (i.e., abnormal sperm forms) have a higher incidence of sperm aneuploidy than those with normal semen parameters [8–11]. Recent observational studies have revealed a possible association between abnormal semen parameters and impaired fertilization rates and embryonic development, even after IVF/ICSI [12]. An emerging concern is the potential link between these conditions and an increased risk of aneuploidy in sperm and embryos.

Molecular genetics is continuously evolving and becoming more advanced. Genetic testing of single cells is now feasible, and the genetic testing of sperm cells is one of the most exciting fields within the ART community. Since 1984, fluorescent *in situ* hybridization (FISH) has been used to analyze aneuploidy rates in human spermatozoa [13]. However, this technique has limitations related to the number of specific chromosomes and the risk of hybridization failure. Next-generation sequencing (NGS), a novel tool in molecular genetics, is the standard method used in ART for the preimplantation genetic testing of embryos for aneuploidy (PGT-A) and monogenic disorders (PGT-M). This method enables the evaluation of entire chromosomes without constraints on the number of accessible cells. NGS has previously been applied to analyzing single sperm cells in known translocation carriers [14, 15]. However, genetic data on human sperm are currently limited and warrant further study. Identifying genetic abnormalities in sperm is crucial for understanding the outcomes of ART and their influence on its success or failure. Examining instances of chromosomal abnormalities in candidate sperm for ICSI is particularly beneficial for understanding the outcomes of both ICSI and embryo development.

The primary objective of this cross-sectional prospective study was to use advanced NGS to comprehensively examine the molecular karyotyping of individual sperm cells and to compare the aneuploidy rates of infertile individuals with severe oligoteratozoospermia and with normal semen. The study was expected to enhance understanding of the genetic etiology behind severe sperm abnormalities to facilitate the development of an individualized approach to reproductive intervention to help more patients build a family.

## Materials and methods

The protocols developed for this study received approval from the Human Research Ethics Committee, Faculty of Medicine Ramathibodi Hospital, Mahidol University (protocol no. MURA2023/25, date: January 12, 2023). Written informed consent was obtained from all patients following the study’s approval by the Institutional Ethics Committee.

### Study population

The study was conducted at Ramathibodi Hospital in Bangkok, Thailand, from 12 January to 30 November 2023. Eligible infertile couples were recruited and separated into two groups based on semen quality. The study group comprised seven individual males with severe oligoteratozoospermia whereas the control group included seven individual males with normal semen according to the 2021 recommendations of the World Health Organization [16]. Severe oligoteratozoospermia was diagnosed when the sperm concentration was below 5 × 10^6^ mL^−1^ and the normal morphology was 4%. In contrast, the control group exhibited a sperm count exceeding 16 × 10^6^ mL^−1^, normal morphology of ≥4%, and normal motility of ≥42%. Patients with azoospermia, testicular sperm retrieval, active genital tract infections, a history of medications that would impair spermatogenesis in the past 3 months, or undergoing cancer treatment were excluded from the study.

### Sample collection

Following a period of sexual abstinence for 2–7 days, semen samples were collected from participants by masturbation in a sterile container following the WHO criteria for semen collection and analysis [16]. After liquefaction, the semen was analyzed in terms of volume (mL), pH, sperm concentration (10^6^ mL^−1^), and motility (%). The sperm morphology was analyzed using the Kruger strict criteria. After analysis, the leftover samples were processed for sperm purity. Spermatozoa were isolated from somatic cells via a gradient centrifugation technique using 90% and 45% Sil-Select STOCK™ solution (Fertipro NV, Belgium) following the manufacturer’s instructions. Centrifugation was performed at 250 g for 10 minutes, and the pellet was rinsed using a flushing medium at 200 g for 8 minutes (Ferticult^TM^, FertiPro, Belgium). Samples from both groups were centrifuged following the same protocol.

### Micromanipulation

Polyvinylpyrrolidone (10% PVP in Ferticult^TM^, FertiPro, Belgium) was used for the immobilization and convenient handling of sperm. Thereafter, a small aliquot of semen was placed into a fertilization medium (Origio, Cooper Surgical, Denmark) in a Petri dish. The sample was processed by using a micromanipulator for ICSI under an inverted microscope (Nikon Eclipse Ti-U, 400X) with an ELWD 40/0.6 NAMC3 lens. Based on the evaluation of an experienced embryologist, a sperm was defined as having a normal morphology according to the following criteria: the head had a smooth oval shape with a well-defined acrosomal region and no vacuole; the midpiece was aligned with the axis of the head, slender, and of similar length to the head; and the tail was uniformly calibrated along its length, thinner than the midpiece, and without abrupt angulations indicating a damaged flagellum. Every single motile sperm with normal morphology was immobilized, aspirated, and dropped separately in sterile phosphate-buffered saline solution per tube. In total, five sperm cells were isolated for each participant with one tube designated for use as a negative control. This is because the average number of embryos obtained from couples typically does not exceed five.

### Whole-genome amplification

Single-cell whole-genome amplification (WGA) was performed in the original tube from the micromanipulation process using the Multiple Annealing and Looping–Based Amplification Cycles method (cat. no. KT110700150, MALBAC^TM^ Single Cell WGA Kit, Yikon Genomics, China). First, protamine was decompacted with proteinase K (cat. no. 19131, Qiagen^TM^, Valencia, CA); then, 5.5 μL of the cell lysis reaction mix (MALBACTM, Yikon Genomics, China) was added to the sample and the mixture was incubated in a thermal cycle at 50°C for 50 minutes, followed by 10 minutes at 80°C. Finally, 30 μL of the pre-amplification mixture (MALBAC^TM^, Yikon Genomics, China) was added to the reaction. Then, after the 10 cycles of the pre-amplification program (3 min at 94°C, 40 s at 20°C, 40 s at 30°C, 30 s at 40°C, 30 s at 50°C, 30 s at 60°C, 30 s at 70°C, 20 s at 95°C, and 10 s at 58°C), 30 μL of the exponential amplification mixture (MALBAC^TM^, Yikon Genomics, China) was added, and the mixture subjected to 19 cycles of exponential amplification (30 s at 94°C, 20 s at 94°C, 30 s at 58°C, and 3 min at 72°C) using a thermal cycler (cat. no. A24811, SimpliAmp^TM^, Thermo Fisher Scientific, USA). The concentrations of the purified products were quantified using a Qubit™ dsDNA High Sensitivity Assay Kit (cat no. Q33231, Invitrogen, Thermo Fisher Scientific, USA).

### Next-generation sequencing

The final product of the WGA process was analyzed by NGS using the commercially available VeriSeq PGS kit-Miseq (cat. no. RH-101-1001, Illumina Inc.). NGS was conducted according to the manufacturer’s instructions. The WGA product was then sequenced using the Illumina MiSeq® sequencing platform (Illumina Inc). Data analysis was performed using BlueFuse Multi software (Illumina, Inc.), which employs the default parameters. The software provides analytical results for samples, which are then compared with reference genomes and presented as graphical reports. These reports are interpreted to identify chromosome abnormalities. To determine copy number variations (CNVs), each aligned read count is assigned to a bin unit delineated by a fixed length of 1 Mb within the genomic sequence. Sperm are diagnosed as aneuploid if their median chromosomal copy number deviates from the default value, as determined by trained technicians. Horizontal green lines above and below indicate chromosomal gain (copy number >1.25) and chromosomal loss (copy number <0.75), respectively. Approximately 0.3–1.0 million raw reads were generated for each sperm. The rate of reads mapped to the genome was approximately 80%. The average depth of sequencing of each genome was 0.02.

### Statistical analysis

The sample size was calculated for the comparison of two independent proportions. Saei et al. [17] reported a sperm aneuploidy rate of 66% in infertile patients with abnormal semen parameters. They needed at least 35 sperm cells per group to detect a 50% reduction in the aneuploidy rate compared to their control group with normal semen parameters. In this study, the type I and II errors were set at 5% and 20%, respectively. The mean and SD or median and interquartile range were used to describe continuous data and the frequency and percentages were used for categorical data. Groups were compared by using Student’s t-test (or quantile regression) for continuous data and by using the chi-squared test (or Fisher’s exact test) for categorical data. P values of <0.05 were considered statistically significant. A mixed-effect linear model was applied to compare the aneuploidy rates of groups. All data analyses were performed by using STATA version 18 (Stata Statistical Software, Release 18, College Station, TX: StataCorp LLC).

## Results

The study included 14 infertile couples, with 7 couples in each group. All had exhibited primary infertility for 1–16 years. The average age of the male participants was 38.14 years, and no significant differences in male age or infertility duration were observed between groups. The female partners of the study and control groups had mean ages of 33.57 and 37.71 years, respectively. The two groups were categorized according to significant differences in the sperm concentration and normal morphology of the semen samples. The total motile sperm count (TMSC) was a variable outcome used to indicate the differing sperm concentrations of the two groups. The data indicated that the percentage of motility did not differ significantly between the two groups. Table 1 summarizes the baseline characteristics of the study and control groups.

**Table 1:** Patients’ baseline characteristics.

Characteristics	Control group (n = 7)	Study group (n = 7)	P-value
Male age, mean (SD)	38.14 (6.57)	38.14 (7.20)	1.00
Female age, mean (SD)	37.71 (4.35)	33.57 (4.31)	0.10
Infertility duration, median (IQR)	4 (1, 8)	3 (1, 4)	0.79
Smoking, n (%)	1 (14.29)	1 (14.29)	1.00
Alcohol intake, n (%)	5 (71.43)	3 (42.86)	0.59
**Semen parameters**			
Volume (ml), median (IQR)	2.50 (1.50, 4.00)	1.80 (1.50, 4.70)	0.64
Concentration (M/mL), median (IQR)	64 (45, 119)	1.10 (1.00, 1.80)	0.04*
Normal morphology ≥4%, n (%)	7 (100%)	0	<0.01**
Motility (%), mean (SD)	62.71 (11.91)	47.14 (25.14)	0.16
TMSC (M), median (IQR)	108.9 (82.60, 134.80)	0.90 (0.40, 3.30)	<0.01*

*P-value by quantile regression

** P-value by Fisher’s exact test

**Fig 1:**
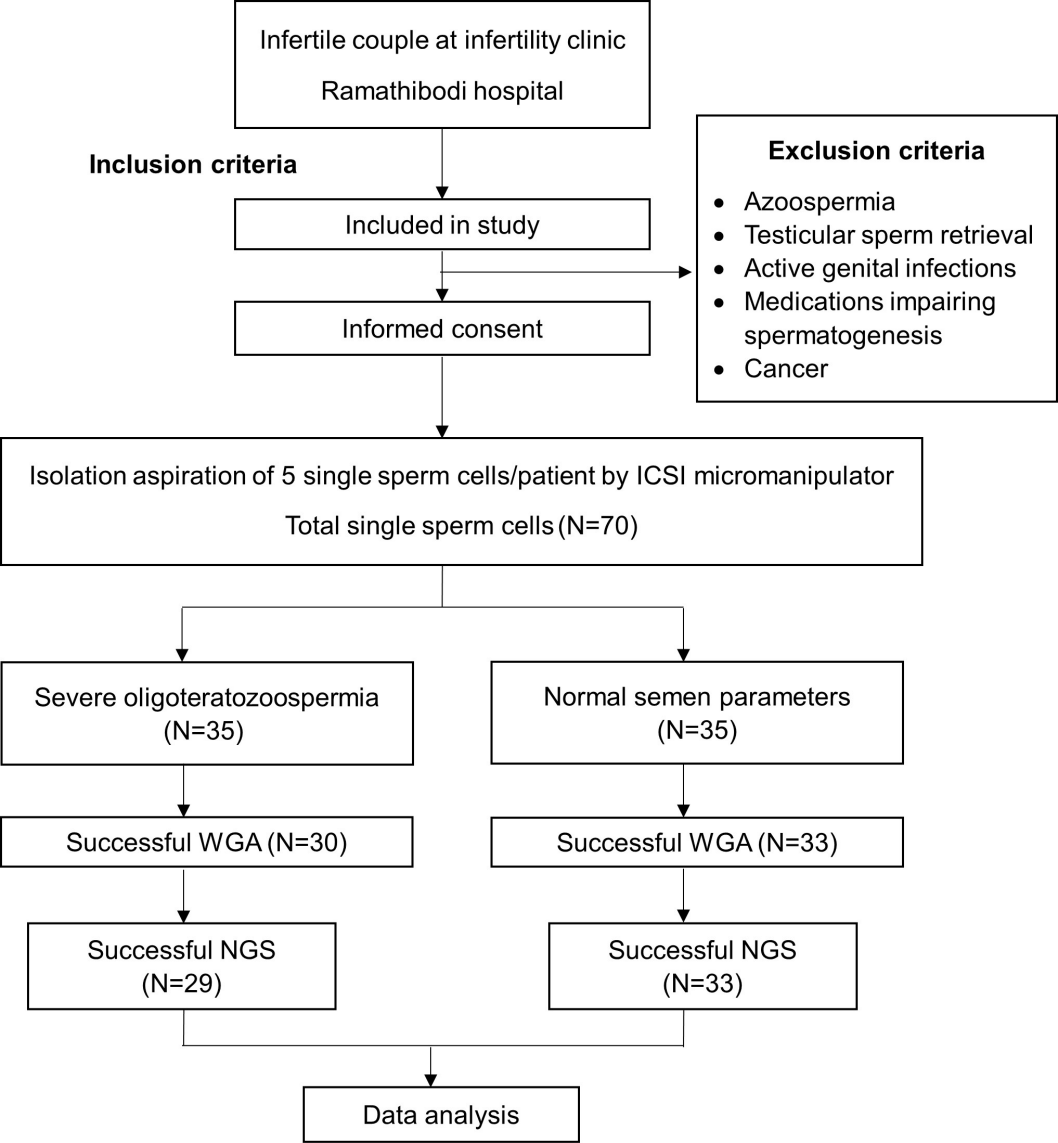
Study flow diagram.

**Fig 2:**
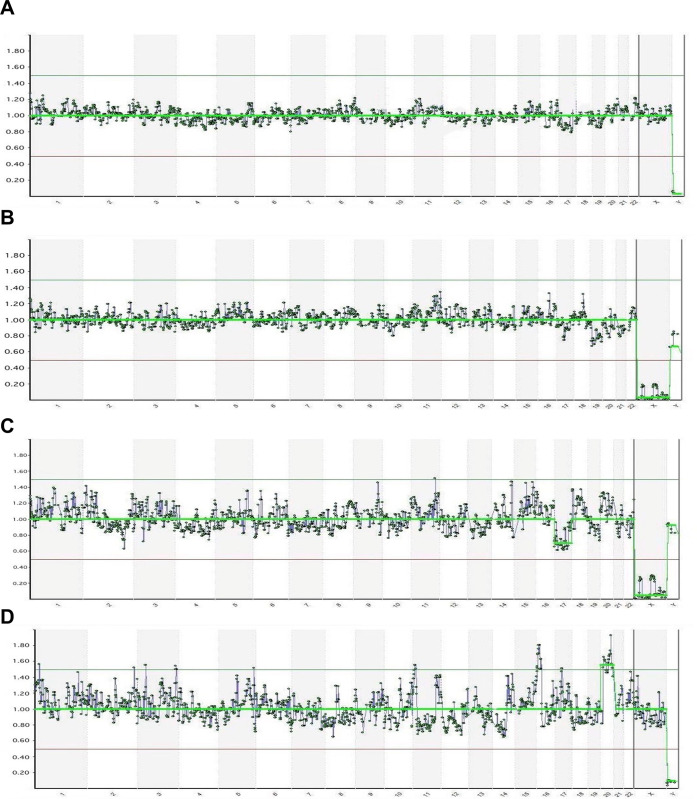
Examples of molecular karyotypes obtained via NGS from a single sperm. (A) 23, X is the normal molecular karyotype from patient C1. (B) 23, Y is the normal molecular karyotype from patient C2. (C) 22, Y exhibiting a single chromosomal loss of -17 in patient S6. (D) 24, X showing a single chromosomal gain of +20 in patient S7.

Seventy individual sperm cells were isolated by micromanipulation, and 63 samples (90%) were successfully amplified by WGA. The process flow is shown in Fig 1. The DNA yield was average and acceptable for each sperm cell. Unfortunately, one sample could not be analyzed by NGS because of technical errors. Consequently, the study and control groups had 29 and 33 sperm cells, respectively, that were analyzed by NGS. Fig 2 presents illustrative examples of molecular karyotypes obtained via NGS from specifically chosen individual sperm cells. All participants in the study group had at least one aneuploid sperm, whereas two of the seven patients in the control group had aneuploidy sperm. Therefore, the study group had a total aneuploidy rate of 25%–75% per patient with an average of 48.28% per pooled sperm whereas the control group had a total aneuploidy rate of 0%–75% with an average of 15.15%. The difference between the two groups was statistically significant (p = 0.007). The aneuploidy rate of single sperm cells was significantly higher in the study group than in the control group, with a threefold increase. The data for the abnormal NGS results are presented in Table 2.

**Table 2:** Aneuploidy rate of morphologically normal single sperm cells.

Based on	Abnormal NGS results		Odds ratio (95% CI)	P-value
	Control group	Study group		
Individual patients	2/7 (0–75%)	7/7 (25–75%)	5.80 (1.30–26.03)	0.02*
Individual sperm cells	5/33 (15.15%)	14/29 (48.28%)	5.23 (1.57–17.32)	<0.01*

* P-value by a mixed-effect linear model

**Fig 3:**
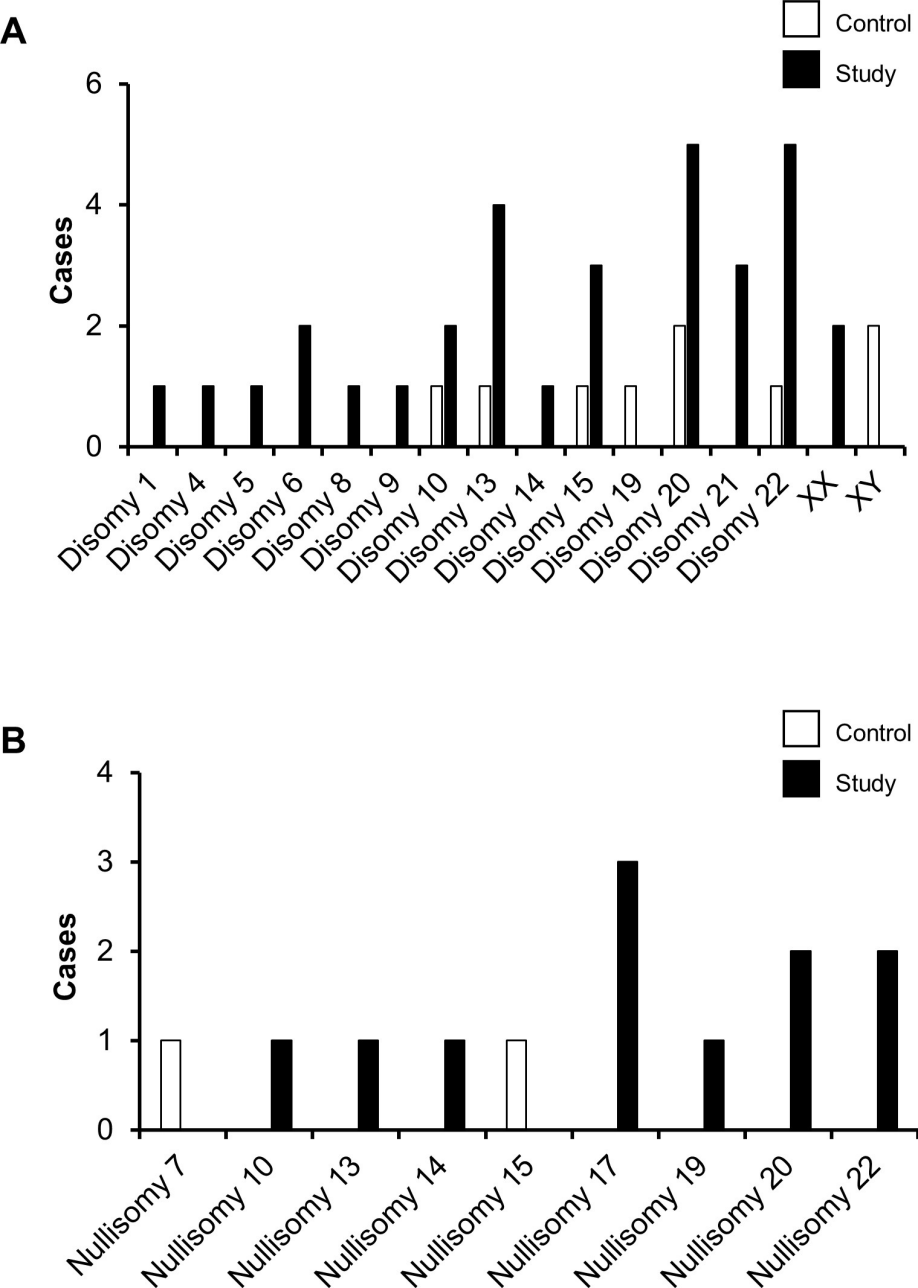
Comparison of chromosomal abnormalities observed in single sperm cells from the control and study groups. (A) chromosome gain (B) chromosome loss.

The study found specific autosomal and sex chromosome abnormalities, including chromosomal disomy and nullisomy. Disomy 20 and 22 were the most frequently observed abnormalities in the study group, with a prevalence of 17.24%. In contrast, disomy 20 was the most commonly observed abnormality in the control group, with a prevalence of 6.06%. Chromosome loss was primarily identified in chromosome 17 in three cases in the study group and chromosomes 20 and 22 in two cases each, as shown in Table 3 and Fig 3. Notably, one sperm may have more than one chromosomal abnormality. Additionally, abnormalities were exclusively found in chromosomes 1, 4, 5, 6, 8, 9, 14, 17, and 21 within the study group. Conversely, no abnormalities were observed in chromosomes 2, 3, 11, 12, 16, or 18 in either group.

**Table 3:** Chromosomal abnormalities observed within single sperm cells.

Abnormalities	Control (n = 33) n (%)	Study (n = 29) n (%)
**Autosomal disomy**		
• Disomy 1	0	1 (3.45)
• Disomy 4	0	1 (3.45)
• Disomy 5	0	1 (3.45)
• Disomy 6	0	2 (6.90)
• Disomy 8	0	1 (3.45)
• Disomy 9	0	1 (3.45)
• Disomy 10	1 (3.03)	2 (6.90)
• Disomy 13	1 (3.03)	4 (13.79)
• Disomy 14	0	1 (3.45)
• Disomy 15	1 (3.03)	3 (10.34)
• Disomy 19	1 (3.03)	0
• Disomy 20	2 (6.06)	5 (17.24)
• Disomy 21	0	3 (10.34)
• Disomy 22	1 (3.03)	5 (17.24)
**Sex chromosome disomy**		
• XX	0	2 (6.90)
• XY	2 (6.06)	0
**Autosomal nullisomy**		
• Nullisomy 7	1 (3.03)	0
• Nullisomy 10	0	1 (3.45)
• Nullisomy 13	0	1 (3.45)
• Nullisomy 14	0	1 (3.45)
• Nullisomy 15	1 (3.03)	0
• Nullisomy 17	0	3 (10.34)
• Nullisomy 19	0	1 (3.45)
• Nullisomy 20	0	2 (6.90)
• Nullisomy 22	0	2 (6.90)

In the sex chromosomes in the sperm cells, 28 of the 62 samples (45.16%) contained the X chromosome, whereas 30 samples (48.39%) contained the Y chromosome. Of the remaining samples, two had the XX configuration in the study group, and two had the XY configuration in the control group (Fig 3). The results indicated no loss of sex chromosomes. Both groups displayed similar rates of sex chromosomal disomic aberration at 6.9% and 6.06%, respectively, and there was no significant difference between them.

To describe the frequency of numerical chromosomal abnormalities, chromosomes were grouped according to size and centromere position. Among all the sperm cells studied, the highest frequency of abnormalities was found in chromosome group D (chromosomes 13–15), accounting for 26% of abnormalities observed (13 of 50) (Table 4). Moreover, 10 sperm cells (16.13%) had at least one abnormality in chromosome group D, as shown in Table 5. The second-most abnormalities were observed in chromosome group F (chromosomes 19–20) and G (chromosomes 21–22) each at 22% (11 out of 50). These abnormalities were found in 10 sperm cells (16.13%) for group F and 9 sperm cells (15%) for group G. Autosomal abnormalities in chromosome groups C, E, B, and A were observed at rates of 18%, 6%, 4%, and 2%, respectively, as shown in Table 4. In the control group, abnormalities were observed in chromosome groups C, D, F, and G. Groups D and F notably exhibited the highest abnormalities, with 6.06% found in 2 sperm cells for each group. In the study group, abnormalities in groups D, F, and G were at 27.59% (8 out of 29), which was almost triple the rate of the control group. The specific details on the chromosome abnormalities of individual sperm cells are provided in Table 6.

**Table 4:** Frequency of autosomal chromosome abnormalities in the control and study groups.

Grouped autosomal chromosomal abnormalities	Total frequency	%
**Autosomal disomy + nullisomy**		
• A (Chromosomes 1–3)	1	2
• B (Chromosomes 4–5)	2	4
• C (Chromosomes 6–12, exclude X chromosome)	9	18
• D (Chromosomes 13–15)	13	26
• E (Chromosomes 16–18)	3	6
• F (Chromosomes 19–20)	11	22
• G (Chromosomes 21–22, exclude Y chromosome)	11	22
**Total**	50	100

**Table 5:** Classification of autosomal abnormalities in sperm cells by specific chromosome groups for each patient.

Group	Patient	Sperm with an abnormal chromosome N (%)	Sperm with an abnormal chromosome classified by specific chromosome groups N (%)						
			A*	B*	C*	D*	E*	F*	G*
Control (n = 33)	C1	0	-	-	-	-	-	-	-
	C2	0	-	-	-	-	-	-	-
	C3	0	-	-	-	-	-	-	-
	C4	2	-	-	-	1	-	1	1
	C5	3	-	-	1	1	-	1	-
	C6	0	-	-	-	-	-	-	-
	C7	0	-	-	-	-	-	-	-
	**Total**	**5** **(15.15)**	**0**	**0**	**1** **(3.03)**	**2** **(6.06)**	**0**	**2** **(6.06)**	**1** **(3.03)**
Study (n = 29)	S1	1	-	1	1	-	-	-	-
	S2	1	-	-	-	1	-	1	1
	S3	2	-	-	1	1	-	2	1
	S4	3	1	-	1	2	-	1	2
	S5	2	-	-	-	1	-	-	2
	S6	2	-	-	1	1	1	1	-
	S7	3	-	1	2	2	2	3	2
	**Total**	**14** **(48.28)**	**1** **(3.45)**	**2** **(6.89)**	**6 (20.69)**	**8 (27.59)**	**3 (10.34)**	**8 (27.59)**	**8 (27.59)**
All (n = 62)	**Total**	**19** **(30.65)**	**1** **(1.61)**	**2** **(3.23)**	**7** **(11.29)**	**10 (16.13)**	**3** **(4.84)**	**10 (16.13)**	**9 (15.00)**

*Chromosome groups according to sizes and centromere positions: A: chromosomes 1–3, B: chromosomes 4–5, C: chromosomes 6–12, D: chromosomes 13–15, E: chromosomes 16–18, F: chromosomes 19–20, and G: chromosomes 21–22.

**Table 6:** Detailed examination of chromosome abnormalities observed in individual sperm cells.

Group	Patient	Sample	Chromosomes		Interpretation
			Gain	Loss	
**Control**	4	3	XY	-	Abnormal
		4	13, 19, 20, 22	15	Abnormal
	5	1	XY	-	Abnormal
		3	15, 20	-	Abnormal
		4	10	7	Abnormal
**Study**	1	3	5, 6	-	Abnormal
	2	2	13, 21, 22	20	Abnormal
	3	3	20	-	Abnormal
		4	10, 15, 20, 21	-	Abnormal
	4	2	20, 22	-	Abnormal
		3	1, 9, XX	13	Abnormal
		4	13, 14, 21, XX	22	Abnormal
	5	1	15, 22	-	Abnormal
		3	22	-	Abnormal
	6	4	-	17	Abnormal
		5	10, 15	19	Abnormal
	7	1	20	-	Abnormal
		2	4, 8, 13	10, 17, 20, 22	Abnormal
		5	6, 13, 20, 22	14, 17	Abnormal

## Discussion

This study was designed to compare the genetic results of single sperm cells with a normal appearance according to different semen parameters. The NGS results showed that the study group had a higher prevalence of chromosomal abnormalities than the control group. Almost 50% of sperm cells from the study group carried abnormal chromosomes, which was three times higher than that of the control group. This finding may help explain the unsuccessful outcomes of some infertile couples in terms of the fertilization rate, cleavage rate, blastulation rate, and aneuploidy rate of embryos, which is essential to treating severe male infertility with ICSI. The results also indicate that abnormal chromosomes in embryos can be generated by severe male infertility.

The high prevalence of abnormal chromosomes is consistent with previous studies that used molecular cytogenetic analysis to examine individual sperm cells in cohorts with abnormal semen parameters. Bernardini et al. [18] used double in situ hybridization and found significantly higher rates of sperm aneuploidy for autosomes 1 and 7 in morphologically normal sperm with a TMSC below 5 × 10^6^. The same trend was observed in several previous studies using FISH, which reported a higher prevalence of aneuploidy compared with the control group, especially in sex chromosomes, in individuals diagnosed with severe oligozoospermia [19, 20], teratozoospermia [21, 22], and oligo-astheno-teratozoospermia (OAT) [23]. However, these earlier studies often reported the overall rate of chromosomal abnormalities in the sample, which made it difficult to accurately predict the effect of the sperm aneuploidy rate on the outcomes of ICSI, which involves selecting only a few sperm cells. The present study was designed to select only sperm cells with excellent morphology as candidates for ICSI.

Furthermore, the technologies used for genetic testing can affect the results. For example, FISH provides fast and straightforward results, and it is suitable for studies that require data on many sperm cells. However, it cannot examine all chromosomes, which limits its scope. Therefore, the prevalence of chromosomal abnormalities in sperm cells from both the study and control groups is a result that has not been observed in a previous study.

In cases of severe male factor infertility where abnormal semen parameters are often present, indications for IVF/ICSI are considered. Most research has shifted toward selecting a smaller number of high-quality sperm cells with normal genetic characteristics. Patassini et al. [24] pioneered using array comparative genomic hybridization (aCGH) to evaluate the aneuploidy rate in single motile sperm cells with normal morphology selected by micromanipulation. They applied a comprehensive screening approach to all chromosomes in single sperm cells and obtained a 7.8% aneuploidy rate in fertile sperm, which indicates that having only normal motile sperm with good morphology does not necessarily confirm genetic normality, even among fertile individuals. Therefore, abnormalities can also be found in infertile patients even when micromanipulation is used to select sperm for ICSI, especially those with severely abnormal semen parameters. Saei et al. [17] reported a sperm aneuploidy rate of 66% in five cases of OAT when using aCGH. This is higher than the rate of 48.28% in the present study, which may be because OAT affects the aneuploidy rate more than severe oligoteratozoospermia. Previous studies [17, 25] on severe male factor infertility using FISH and aCGH found more significant abnormalities in chromosome groups D, F, and G (i.e., chromosomes 13, 21, and 22), which is consistent with the results of the present study obtained by NGS as given in Table 5. Our study emphasizes that this consistency strengthens the validity and robustness of the observed abnormalities across different methodologies.

A recent previous study that used whole-genome sequencing to analyze aneuploidy in individual sperm cells reported that sperm cells with aneuploid autosomes exhibited fewer crossovers on average than normal cells [26]. Chromosome group G is associated with an increased risk of achiasmate chromosomes that are characterized by a single recombination site. This trait may lead to the loss of protective mechanisms against the premature segregation of homologous chromosomes, ultimately resulting in nondisjunction during gamete formation [27]. Moreover, studies have found that recombination errors can lead to abnormal chromosome segregation as well as meiotic arrest, resulting in partial blocks in some germ cells [28]. Meiotic arrest in turn results in abnormal sperm production; this is one cause of severe oligoteratozoospermia. Studies on chromosome group F (chromosomes 19 and 20) are limited, mainly owing to the challenges associated with FISH; however, our study findings were consistent with those of previous studies on patients with oligoasthenoteratozoospermia [17]. Over time, there may be significant changes in sperm aneuploidy levels in some individuals, indicating a susceptibility to fluctuations in aneuploidy frequencies. These changes may be caused by temporary factors or lifestyle adjustments, affecting sperm aneuploidy independently of errors during spermatogenesis. Despite documented associations between increased risks of disomy 13 with exposure to cigarette smoke [29] and gonosome aneuploidy with alcohol consumption [30], understanding these correlations is difficult because of the small sample size in this study. Finally, smaller and/or acrocentric chromosomes may be linked to sperm aneuploidy and abnormal chromosome segregation, suggesting that smaller chromosomes play a role in the development of aneuploidy. Saei et al. [17] reported a high rate of sex chromosome disomy in OAT patients at 26.6% of samples, which they obtained by aCGH analysis. This finding contrasts with the present study, in which a different sampling method was employed to minimize the chance of two sperm cells being included in a single sample. In the present investigation, the overall aneuploidy rate of sex chromosomes was 6% in the study group, which did not differ from that in the control group. This study provides valuable insights into the association between severe oligoteratozoospermia and the occurrence of abnormalities, particularly aneuploidy, in sperm with normal morphology. On comparing the members of this group with infertile men with normal semen, we can determine the specific impact of severe semen abnormalities on the genetic integrity of sperm. The consistent use of the same embryologist team and laboratory protocol makes the findings more reliable, enabling a more straightforward comparison with previous studies. Consequently, the results highlight the increased risk of encountering aneuploid sperm in individuals with severe oligoteratozoospermia, emphasizing the need for targeted interventions in assisted reproductive technologies to reduce potential genetic risks.

In a retrospective cohort study, Grammatis et al. [6] explored the impact of abnormal semen parameters on embryos in donor egg cycles by using preimplantation genetic testing for aneuploidy (PGT-A). Their results indicated that abnormal semen parameters had a detrimental effect on the fertilization rate, blastulation rate, and attainment of euploid embryos. Notably, patients with severe oligozoospermia exhibited the lowest blastulation rate of 32.4%. Their findings align with those of the present study, which indicates a clear negative correlation between sperm aneuploidy and embryo development. It can be inferred that embryos with normal genetic components are the only ones likely to successfully progress to the blastocyst stage. However, Mazzilli et al. [31] conducted an observational study on 1,219 ICSI cycles and found no difference in euploid and live birth rates among male factor groups. Future research will require additional data on ICSI outcomes to facilitate a comprehensive investigation into the influence of male factors on euploid rates and obstetric outcomes.

In this study, the control group comprised infertile couples with issues unrelated to abnormalities in semen parameters or non-male factor infertility. The rationale was to assess the effectiveness of sperm selection based on motility and morphology within a cohort exhibiting normal semen characteristics. In addition, the control group represented individuals who may benefit from future IVF/ICSI procedures. The results showed that, while micromanipulation may not eliminate the risk of choosing genetically abnormal sperm, the likelihood of encountering abnormalities in the control group was still lower than that in the study group.

The main contribution of this study was in the application of NGS to the molecular karyotyping of selected sperm cells and mimicking the ICSI process to observe the effects of abnormal semen parameters. However, this study was limited by the complex process for genetic examination of single sperm cells, which relies on specialized techniques and multiple steps. Although WGA was used to study single sperm cells, approximately 10% of the samples could not be amplified and thus could not be used for analysis. This may be due to the densely packed DNA in sperm cells. Future studies may require modified methods to dissolve the head and achieve better amplification efficacy. In addition, this study was limited by the lack of chromosome karyotyping or Y chromosome deletion analysis for each male participant. This data gap is because Ramathibodi Hospital does not routinely conduct genetic evaluations for every case of severe oligozoospermia before ICSI and such procedures are not covered by insurance. Therefore, most couples facing male factor infertility may receive counseling on the risk of aneuploidy and PGT embryo options after ICSI.

The results showed that participants in the study group exhibited a sperm aneuploidy rate of 48%, which was up to three times higher than the rate observed in the control group. However, at least one sperm with normal genetic material was obtained from all participants through the micromanipulation technique regardless of their group. Therefore, the applicability of ICSI was not limited to these specific couples, and it was concluded to remain a viable option to assist infertile couples with severe male factor issues in achieving pregnancy. Effective counseling on the aneuploidy rate, which is potentially higher than that of the general infertile population, and guidance on preimplantation genetic testing are recommended to ensure the transfer of embryos with normal chromosomes.

## Conclusions

Individuals with severe oligoteratozoospermia showed a high sperm aneuploidy rate, even when sperm were selected by the micromanipulation technique. Comprehensive counseling is advised to address the elevated aneuploidy rate, which is potentially higher than that of the general infertile population. Guidance on preimplantation genetic testing is also recommended to ensure the transfer of embryos with normal chromosomes.

## Supporting information

S1 Dataset (PDF)
