# Supplementary material for: Chromosomal analysis of single sperm cells from infertile couples with severe oligoteratozoospermia: A cross-sectional prospective study
Source: PLoS One. 2024 Jun 14;19(6):e0303350. doi: 10.1371/journal.pone.0303350 (PMC11178158; doi:10.1371/journal.pone.0303350)
Supplement: S1 Dataset — (PDF) [file pone.0303350.s001.pdf]

Supporting information

S1 Dataset

Group	Subject	Sample	QC	Result by BlueFuse Multi	Conclusion	Total reads
C	1	1	Pass	23, X	Normal	1401641
C	1	2	Fail	none	None	
C	1	3	Pass	23, X	Normal	1259190
C	1	4	Pass	23, X	Normal	1144767
C	1	5	Pass	23, X	Normal	996555
C	2	1	Pass	23, Y	Normal	1009933
C	2	2	Pass	23, Y	Normal	914897
C	2	3	Pass	23, Y	Normal	935498
C	2	4	Pass	23, Y	Normal	1214949
C	2	5	Pass	23, Y	Normal	1204368
C	3	1	Pass	23, X	Normal	1044315
C	3	2	Pass	23, X	Normal	927383
C	3	3	Pass	23, Y	Normal	1079438
C	3	4	Pass	23, Y	Normal	960754
C	3	5	Pass	23, Y	Normal	1125243
C	4	1	Pass	23, Y	Normal	891635
C	4	2	Pass	23, Y	Normal	1293649
C	4	3	Pass	24, XY	Abnormal	872426
C	4	4	Pass	27, Y (+13, -15, +19, +20, +22)	Abnormal	1239654
C	4	5	Pass	23, X	Normal	961096
C	5	1	Pass	24,XY	Abnormal	1177313
C	5	2	Pass	23, X	Normal	1222790
C	5	3	Pass	25, X (+15, +20)	Abnormal	848623
C	5	4	Fail	none	None	
C	5	5	Pass	23, X (-7, +10)	Abnormal	963073
C	6	1	Pass	23, X	Normal	1273601
C	6	2	Pass	23, X	Normal	1124048
C	6	3	Pass	23, X	Normal	897127
C	6	4	Pass	23, Y	Normal	539488
C	6	5	Pass	23, Y	Normal	1251711
C	7	1	Pass	23, X	Normal	1322783
C	7	2	Pass	23, X	Normal	1205252
C	7	3	Pass	23, X	Normal	1243464
C	7	4	Pass	23, Y	Normal	891635
C	7	5	Pass	23, X	Normal	672136

Group	Subject	Sample	QC	Result by BlueFuse Multi	Conclusion	Total reads
S	1	1	Pass	23, X	Normal	1210180
S	1	2	Pass	23, Y	Normal	1104831
S	1	3	Pass	25, Y (+5, +6)	Abnormal	526139
S	1	4	Pass	23, X	Normal	1061370
S	1	5	Pass	none	None	339420
S	2	1	Pass	23, Y	Normal	363222
S	2	2	Pass	25, Y (+13, -20, +21, +22)	Abnormal	351921
S	2	3	Pass	23, X	Normal	306694
S	2	4	Fail	none	None	
S	2	5	Fail	none	None	
S	3	1	Pass	23, Y	Normal	1078698
S	3	2	Pass	23, Y	Normal	1576104
S	3	3	Pass	24, Y (+20)	Abnormal	563093
S	3	4	Pass	27, X (+10, +15, +20, +21)	Abnormal	452656
S	3	5	Fail	none	None	
S	4	1	Pass	23, Y	Normal	539433
S	4	2	Pass	25, X (+20, +22)	Abnormal	514035
S	4	3	Pass	25, XX (+1, +9, -13)	Abnormal	399130
S	4	4	Pass	26, XX (+13, +14, +21, -22)	Abnormal	429478
S	4	5	Fail	none	None	
S	5	1	Fail	none	None	
S	5	2	Pass	25, X (+15, +22)	Abnormal	1022251
S	5	3	Pass	23, Y	Normal	1091253
S	5	4	Pass	24, Y (+22)	Abnormal	759752
S	5	5	Pass	23, Y	Normal	1038489
S	6	1	Pass	23, Y	Normal	1251751
S	6	2	Pass	23, Y	Normal	1019901
S	6	3	Pass	23, X	Normal	1305448
S	6	4	Pass	22, Y (-17)	Abnormal	1204535
S	6	5	Pass	24, X (+10, +15, -19)	Abnormal	1169969
S	7	1	Pass	24, X (+20)	Abnormal	1245517
S	7	2	Pass	22, Y (+4,+8,-10,+13,-17, -20, -22)	Abnormal	376173
S	7	3	Pass	23, Y	Normal	1177313
S	7	4	Pass	23, X	Normal	1222790
S	7	5	Pass	23, X (+6, +13,-14,-17, +20, +22)	Abnormal	580800
